# Age-related changes of the retinal microvasculature

**DOI:** 10.1371/journal.pone.0215916

**Published:** 2019-05-02

**Authors:** Nikita V. Orlov, Cristopher Coletta, Freekje van Asten, Yong Qian, Jun Ding, Majd AlGhatrif, Edward Lakatta, Emily Chew, Wai Wong, Anand Swaroop, Edoardo Fiorillo, Alessandro Delitala, Michele Marongiu, Ilya G. Goldberg, David Schlessinger

**Affiliations:** 1 Laboratory of Genetics & Genomics, National Institute on Aging/National Institutes of Health, Baltimore, Maryland, United States of America; 2 Division of Epidemiology and Clinical Applications, National Eye Institute/National Institutes of Health, Baltimore, Maryland, United States of America; 3 Neurobiology, Neurodegeneration and Repair Laboratory, National Eye Institute/National Institutes of Health, Baltimore, Maryland, United States of America; 4 Laboratory of Cardiovascular Science, National Institute on Aging/National Institutes of Health, Baltimore, Maryland, United States of America; 5 Istituto di Ricerca Genetica e Biomedica, Consiglio Nazionale delle Ricerche (CNR), Monserrato, Cagliari, Italy; 6 Department of Clinical and Experimental Medicine, Azienda Ospedaliero Universitaria di Sassari, Sassari, Italy; University of Florida, UNITED STATES

## Abstract

**Purpose:**

Blood vessels of the retina provide an easily-accessible, representative window into the condition of microvasculature. We investigated how retinal vessel structure captured in fundus photographs changes with age, and how this may reflect features related to patient health, including blood pressure.

**Results:**

We used two approaches. In the first approach, we segmented the retinal vasculature from fundus photographs and then we correlated 25 parameterized aspects ("traits")—comprising 15 measures of tortuosity, 7 fractal ranges of self-similarity, and 3 measures of junction numbers—with participant age and blood pressure. In the second approach, we examined entire fundus photographs with a set of algorithmic CHARM features. We studied 2,280 Sardinians, ages 20–28, and an U.S. based population from the AREDS study in 1,178 participants, ages 59–84. Three traits (relating to tortuosity, vessel bifurcation number, and vessel endpoint number) showed significant changes with age in both cohorts, and one additional trait (relating to fractal number) showed a correlation in the Sardinian cohort only. When using second approach, we found significant correlations of particular CHARM features with age and blood pressure, which were stronger than those detected when using parameterized traits, reflecting a greater signal from the entire photographs than was captured in the segmented microvasculature.

**Conclusions:**

These findings demonstrate that automated quantitative image analysis of fundus images can reveal general measures of patient health status.

## Introduction

In our aging society age-related diseases and disorders pose increasing health concerns. Cardiovascular disease remains the leading cause of mortality worldwide and thus the status of our vasculature is of great clinical importance. Large studies have detailed aging- and metabolic-related changes in the status of the heart and circulatory system, including blood pressure, pulse-wave velocity, intima-media thickness, and electrocardiogram and echocardiogram parameters. What happens at the level of smaller vessels has been more difficult to determine; but because of its unique accessibility, the microvasculature of the eye has become an actively studied source of information.

In contrast to expert recognition of a pathognomonic gestalt, detailed examination and quantitative analysis of parameterized features of blood vessels in the eye requires segmentation; that is, the microvasculature must be extracted and visualized. For example, Niemeijer [1, 2] conducted a study showing that manual segmentation by an experienced ophthalmologist provides accurate results. Implementing various segmentation protocols, ophthalmologists have progressively characterized features of caliber and morphology of the retinal microvasculature, including tortuosity (curvature), fractal dimension (self-similarity of levels of capillary size), and length and number of branches and segments of capillaries [3].

Using a suite of tortuosity measures, including various measures of the integral curvature along each blood vessel [4, 5], several groups have reported [3] suggestive correlations of parameterized traits with conditions and diseases. For example, retinal arteriolar tortuosity has been observed to be associated with early kidney dysfunction in type 1 diabetes [6], as well as with blood pressure and cardiovascular risk factors [7].

Fractal dimension as a measure of self-similarity of the retinal blood network has also been considered a potential biomarker for disease detection. One implementation of the Fractal dimension algorithm, Block counting across different scales of the image has become a popular method for estimating the self-similarity at different levels of complexity, with certain ranges of fractal dimension associated with blood pressure [8] and cognitive dysfunction [9]. Bifurcations in the vessel maps have also been explored for effects of age [10], cardiovascular conditions [11], and Alzheimer’s disease [12].

The interest in such analyses is further motivated by the attractiveness of possible semi-automated computer-assisted tools for assessment and diagnosis of eye-related pathology. Although regular expert screening and timely treatment are required for diseases like diabetic retinopathy [6], massive screenings pose a challenge of workload for professional ophthalmologists. Successful examples of advanced screening include automated differentiation of drusen, exudates and cotton-wool spots [2], identification of reticular pseudodrusen [13], and age-related macular degeneration [14].

In many of the studies thus far, the sizes of cohorts have been small, and the effects of aging per se has not been studied in detail. Aging and age-related disorders are intertwined, and in order to better understand pathogenesis we need to analyze aging-associated microvascular changes. A recent publication [15] has applied powerful artificial intelligence methods to demonstrate conclusively that the age of an individual can be well estimated from fundus photographs, and that fundus photograph features can be correlated with several cardiovascular conditions. And in another recent study of 1,187 participants in the Australian Heart Eye Study (16), proprietary SIVA software provided strong further support to the association of several retinal vessel measures with more diffuse and severe coronary artery disease. However, a systematic assessment of aging-related changes in specific features of retinal microvasculature has not been investigated on a population basis.

In this study, we aimed to extend the quantitative analysis of the extent of age-related changes in segmented retinal microvasculature using eye images from the SardiNIA [16] longitudinal study and from the Age-Related Eye Disease Study (AREDS) clinical trial [17], which was designed to explore the effect of zinc and anti-oxidant vitamin supplements on age-related macular degeneration in a cohort assembled from the American population. We implemented an automated approach in which the segmented vessel map was used as an input to automated computation of traits, based on tortuosity, fractal measures and analysis of junction for vessel elements. We detected several traits correlated with age and tested for any correlation with a number of cardiovascular variables in the same individuals. Finally, we also compared the detection of an age-dependent signal by a set of algorithms designed to compare overall images with the results for the parameterized measures.

## Methods

The SardiNIA study is approved by Ethics Committee ASL1 Sassari, Italy as protocol 2171/CE for “Genetics and Epidemiology of conditions associated with aging in the Sardinian population (SardiNIA)”. The Ethical Committee approval covers documents including the “Informed Consent for genetic research”; the “Informed Consent for research on enumerated traits in the study protocol 2171/C”; Informed Consent for Clinical Research; and the “Protocol for the Study”. The AREDS study was approved by the National Eye Institute IRB for Human Subjects for this Multi-center study. Written informed consent was obtained from each participant after explanation of the nature and possible consequences of the study.

**Fig 1:**
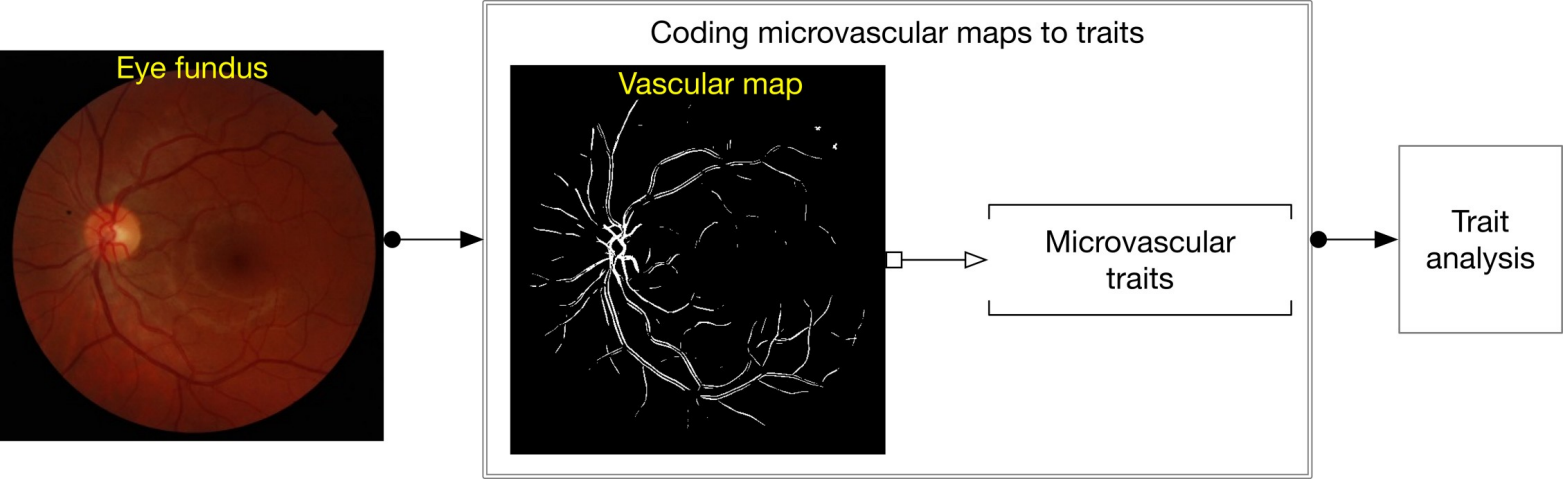
Schematic diagram for analyzing vascular maps.

Abramoff et al [18], extensively review procedures for fundus image analysis, requiring segmentation of the vessel as an important part of the analysis of microvasculature and also for the detection of other abnormalities of the eye (hemorrhage, neovascularization, drusen, pigmentation abnormalities, melanoma, etc.). Segmentation is followed by tracking of the branches to produce connected lines/curves out of the black and white pixel map resulting from the segmented map. We employed a fully automated approach that included segmentation of vessel maps as a first step, followed by computation of vessel traits of potential interest to ophthalmologists (see Fig 1). We refer to them as microvascular traits.

### Cohorts and fundus photographs

The data are digital RGB color images of eye fundus. We used two data sets: SardiNIA [16] and AREDS [17]. The participants in SardiNIA study were recruited from residents in a cluster of four towns in the Ogliastra region of the island of Sardinia, starting in 2001 with repeated assessments every three years; the participants ranged from 20.3 to 88.4 years old [16]. Of over 7,000 images collected at the fourth visits (2011–2014), we selected for analyses 4,552 images from 2,280 participants that yielded clear segmentation maps.

AREDS (Age-Related Eye Disease Study) is a data set, initiated by National Eye Institute as a clinical trial. Participants are US-based population. We used 33,439 [19] images corresponding to the macular region of the right eye (30-degree images) taken over the course of the study and further narrowed the data to a subset including “category 1, controls” and “category 1, others”–that is, restricting the sample to those images judged not to have AMD. This resulted in a total of 7,949 images corresponding to 1,178 participants ranging from 59 to 84 years old. There was no further selection based on quality assessment of images in this set. The age distributions were centering at 49.7 years old for SardiNIA and 68.8 for AREDS (S1 Fig).

### Segmentation of vessel map

We used Soares mlvessel-1.4 software [20] to segment the vessel maps from eye images. The total microvasculature, including both venules and arterioles, were extracted and considered together in subsequent analyses. We implemented a fully-automated method for evaluation of vessel thickness derived from the segmented eye vessel map. To fit the image into the training images used by the mlvessel-1.4, which were all 565×584 pixels, we downscaled our fundus images from the original 3872×2592 to 565×584 pixel size (a downscale factor ~ 5.5). With this approach, the accuracy of caliber evaluation relied heavily on the accuracy of segmentation, which was dependent on the automated program. The program did not distinguish between venules and arterioles, but estimated the caliber of all vessel branches in the whole set of eye images and computed the average caliber (uber-mean, Um). We then considered two categories: less than F*Um (“Thin”) and greater than F*Um (“Thick”). The vessel calibers of the whole data sample cover a total range of Thin and Thick vessels well, we used a value of the parameter F of 0.5, a value at which the majority of vessels were thereby scored as Thick in our analyses. We also considered a third category comprising vessels of all calibers taken together (that is, “Total microvasculature”), and as expected, the results were similar to the analysis focused on Thick vessels (see text).

### Ophthalmology-specific traits

We implemented a brief library of ophthalmology-specific traits composed of three families, 2D Tortuosity, Fractal features and Junction statistics. The relevant MATLAB scripts (https://github.com/orlovni/SardiNIA-retinograms) are open for free public use with no restriction under GNU GPL software license.

#### Tortuosity traits

We adapted the set of seven two-dimensional tortuosity features proposed in [4, 5] (Table 1; Part A of S1 Appendix). The set comprises the ratio of a curve's arc and chord lengths (T1); total curvature over a whole segment (T2); total squared curvature over the segment (T3); total curvature normalized to a curve's arc length (T4); total squared curvature normalized to a curve's arc length (T5); total curvature normalized to a curve's chord length (T6); and total squared curvature normalized to a curve's chord length (T7).

**Table 1:** Definition of microvascular traits.

Trait	Full name
	***Tortuosity family (as in Part A of S1 Appendix)***
t1 (t2)	Ratio of curve's arc length and chord length, sampled (interpolated)
t3 (t4)	Total curvature, sampled (interpolated)
t5 (t6)	Total squared curvature, sampled (interpolated)
t7 (t8)	Total curvature normalized by arc length, sampled (interpolated)
t9 (t10)	Total squared curvature normalized by arc length, sampled (interpolated)
t11 (t12)	Total curvature normalized by chord length, sampled (interpolated)
t13 (t14)	Total squared curvature normalized by chord length, sampled (interpolated)
t15	Interpolated arc length / sampled arc length
	***Fractal dimension family (as in Part B of S1 Appendix)***
f2	Box counts, scale 2
…	…
f8	Box counts, scale 8
	***Junction family***
j1	Number of terminal points
j2	Number of bifurcation (forking)points
j3	Number of crossing points

The vessel curve is defined by a set of anchor points sampled by the program. Tortuosity measures were calculated based on those points. Because capture of portions of the vessel map may be noisy, we also implemented a smoothing approximation of the branch contour for each of the computed tortuosity traits (T1-T7). Thereby, each trait becomes a pair of values corresponding to 'sampled' and 'smoothed' contours: T1 gives rise to t1(sampled) and t2(smoothed); T2 splits to t3, t4, and so on. Additionally, we computed the ratio of smoothed vs. sampled values for the arc length (T8, corresponding to t15 in Figures and Tables), bringing the total number of assessed tortuosity traits to 15. Details are given in Table 1 and Part A of S1 Appendix.

#### Fractal features

We employed a box count approach [21], in which seven per-scale box counts in each of seven scales were used for estimating self-similarity in each scale. The software employed for the box counting was by Alceu Costa [22]. Fractal traits (f2, f3, …, f8) were per-scale box counts on a log scale divided by resolution on a log scale (details in Part B of S1 Appendix).

#### Junction analysis

The concept of junction analysis comes from forensic research, where it is applied to fingerprints [23]. For this analysis we counted the number of terminal points in a vessel map skeleton (j1), as well as the number of bifurcations (j2), and number of terminal points (j3). We used the concept of “number of crossing points” and an eight-connection ridge flow pattern [24] on skeleton image analysis of the junction patterns, covering the entire image with a 3x3 pixel window.

### CHARM features

CHARM features present a large set (2,919) of generic image descriptors that permit the comparison of any type of images. CHARM features provide a tool for a broad range of biological, biometrical and biomedical applications [25]. The feature library contains diverse algorithms [26], including polynomial coefficients (Zernike, Chebyshev, Chebyshev-Fourier), multi-scale histograms, features derived from the Radon transforms and Gabor filters, Haralik and Tamura texture algorithms, fractals, edge and object statistics. For each given pixel plane the features are computed from the raw pixels, as well as from the set of transforms (Fourier, Chebyshev and wavelets) and second-order transforms. CHARM features (https://github.com/wnd-charm/wnd-charm) are open for free public use with no restriction under GNU GPL software license.

### Analyzing traits and features for classification accuracy

Our goal is finding aging patterns in fundus images. Classification is a powerful tool in pattern recognition, in which an algorithm learns categories (e.g., defined age ranges and corresponding data values for the individuals in each category) in a constrained training set and then predicts the category of samples that had not been included in the training set; the degree to which the class of an unknown sample can be correctly predicted is a measure of the reliability of the analysis for the data given and the classifier used.

Each image-sample (image) was processed into 75 microvascular traits–that is, 25 for Thick elements, 25 for Thin, and 25 for Total microvasculature, as outlined above. For the alternative analyses each image was processed for 2,919 CHARM features. For both analyses (traits or feature types) the initial feature space was reduced to a smaller most discriminating subset to enter the classification stage. The space reduction for both traits and features used three independent selection algorithms along with two different classification methods. One classifier was WND [26] using Fisher scores that assess the top 75 features for use in a classification analysis. The other two classifiers were the SVM algorithm [27], combined with minimal redundancy and maximal relevance [28] feature reduction, and SVM with conditional mutual information minimization [29]. For both SVM methods we used the top 30 traits or features in classification analyses.

We used standard non-exhaustive twofold cross validation with 10 randomized splits of the images into training and test sets. In our cross-validation we restricted consecutive eye images from the same participant to be either in the training or in the test set, not in both. The reported accuracy was the frequency of correct calls of the age cohorts, using the average of the three classifiers, the average of the two (or more) age classes into which the cohort sample was divided, and the average of the ten random splits.

## Results

### Assessment of trait values in segmented microvasculature

We used the mlvessel-1.4 segmentation program of Soares [20] to extract microvasculature from standard retinal photographs for 2,280 SardiNIA population cohort participants and 1,178 AREDS control participants; randomly chosen representative photos and segmented profiles from the SardiNIA population cohort are in S2 Fig. We carried out automatic scoring of a set of 25 traits on each image. The traits were subdivided into 3 classes (see Methods): 15 traits examining features of the curvature of microvasculature vessels, 7 looking at fractal self-selection properties, and 3 measuring the numbers of junctions of microvasculature elements. We measured trait values for the entire set of microvasculature elements detected by the program (Total microvasculature) and separately for the Thick elements and Thin elements, defined as greater and less than the rough caliber threshold described in Methods. However, we found that the bulk of the vessels were of the Thick class, and the trait measures for Thin branches were very noisy (see Discussion). Consequently, little if any additional information or statistical power was gained by analyzing the Thin vessels alone or using the Total microvasculature. Therefore, we continued by focusing on the most abundant and relatively well segmented Thick class of blood vessels.

We excluded trait j3 (number of crossing points of vessels) from analyses, because it gave no informative value in age discrimination. On further examination, j3 appeared to be a “phantom trait”; unlike bifurcations (j2) and terminal points (j1), the apparent crossings were just overlapping (e.g. projections) of two distinct vessels.

**Fig 2:**
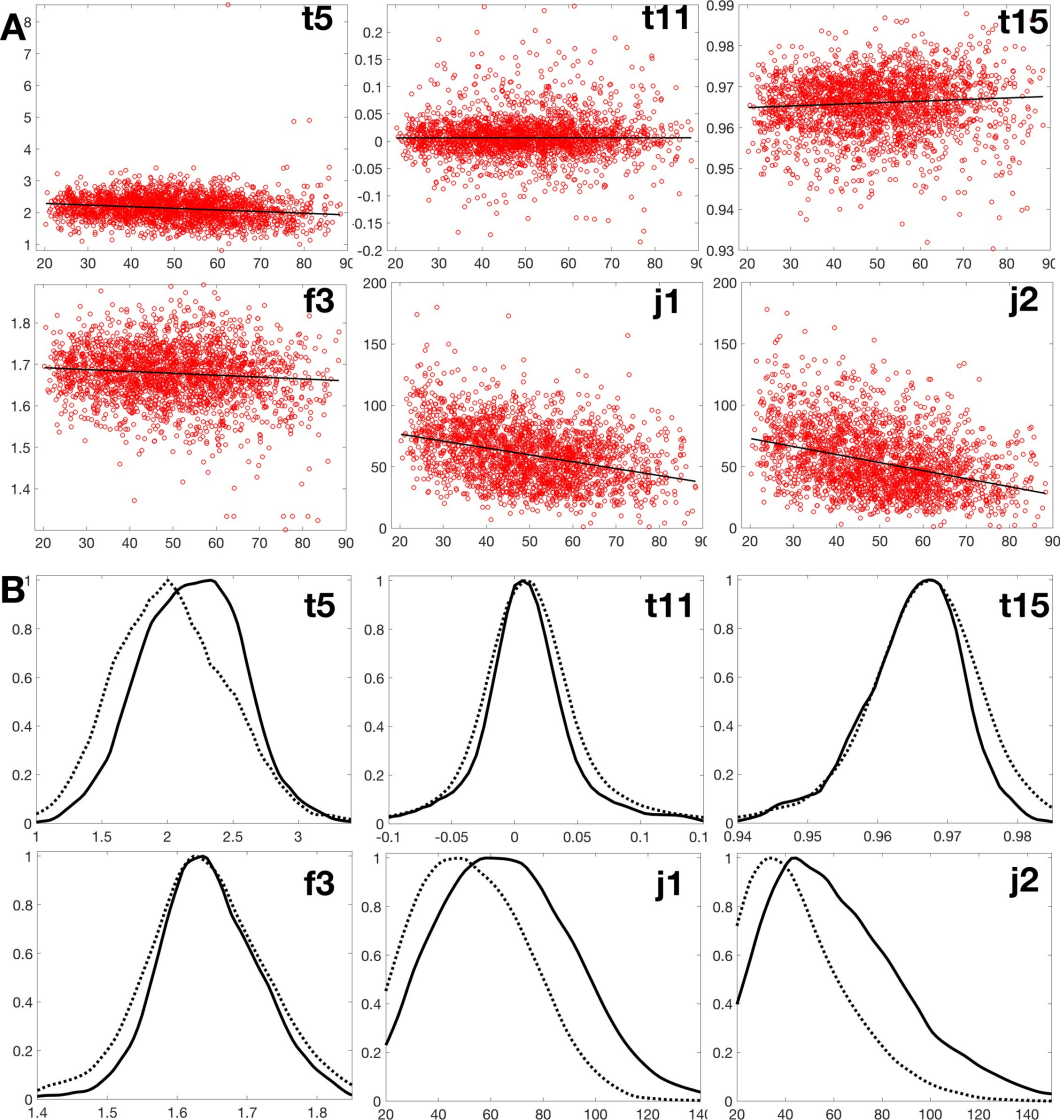
SardiNIA set. (A) Distribution of six selected microvascular traits according to participants’ age. The ordinates are, for t5, total square curvature; j1 and j2, numbers of terminal points and bifurcations; t11, t15, and f3, dimensionless ratios. (B) Histograms of the same six traits, with numbers of individuals as ordinate and the y-axis measures of panel A on the abscissa (solid: <50 y.o., dashed: >50 y.o.).

**Fig 3:**
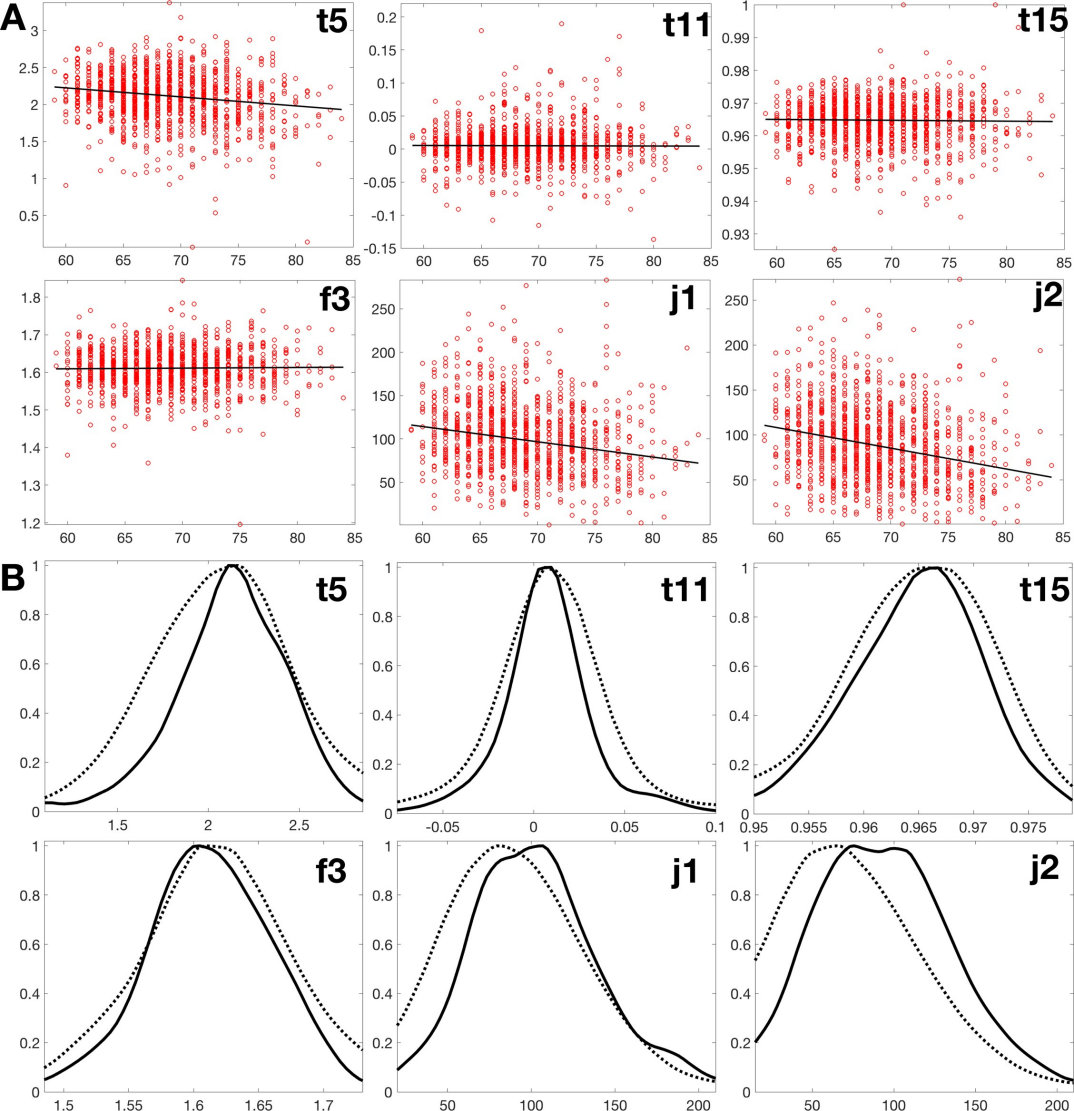
AREDS set. (A) Distribution of the same six microvascular traits as in Fig 2, ordinates and abscissas as in Fig 2. (B) Histograms (solid: <68.8 y.o., dashed: >68.8 y.o.).

Each participant contributes only one image (this is true for all analyses but classification). The values for each trait (24 traits in total, with j3 removed) are plotted as a function of age for the Sardinians and the AREDs cohort (S3 Fig and S4 Fig; also Figs 2A and 3A). By visible inspection, four traits (t5, f3, j1, and j2, boxed in rectangles in the Figures) showed an apparent change with age in SardiNIA, and on further analysis were the only ones that showed a significant slope with age. Three of those four (t5, j1, and j2) showed the most evident trend in AREDS as well. We analyzed those further along with two traits, (t11, t15, circled in S3 Fig and S4 Fig), chosen randomly from among those that showed no apparent age trend. The effect of age on t5, j1, j2, and f3 is also evident in distributions of values for the six traits, represented in Figs 2B and 3B for younger and older subgroups of each cohort defined by age above or below the median population age.

Because of the different age range in the two cohorts, the younger and older subgroups were defined according to a different cutoff age (see S1 Fig). In the SardiNIA cohort, the younger and older subgroup was below and above 50 years of age, respectively. In AREDS, the cutoff for the younger and older subgroup lay at 68.8 years of age. A shift to lower values with higher age was obvious for traits j1 and j2 in both cohorts.

Quantifying the effect of age, Table 2 gives results for SardiNIA data for the overall population (males + females). Instead of p-values we show the p-values adjusted for false discovery rate (FDR) [30]. As expected, no significant difference was seen for the two “control” traits, t11 and t15 (p > 0.05). In contrast, the four strongly affected traits all showed a strong negative trend with age. The two strongest Junction traits were the number of bifurcations (j2, adjusted p-value ~1.8e-53) and the number of terminal points (j1, adjusted p-value ~2.1e-45). In the SardiNIA cohort we see a steady decline of the j2 value from 74 to 31 (~47%) and of the j1 value from 77 to 41 (~58%), over the age range of 20.3 to 88.4 years (S1 Table).

**Table 2:** Correlation with age for SardiNIA microvascular traits.

Microvascular trait, Thick	Pearson	p-values adjusted
Number of bifurcation points (j2)	-0.318	1.8E-53
Number of terminal points (j1)	-0.293	2.1E-45
Total squared curvature (t5)	-0.144	3.7E-11
Scale 3 fractal (f3)	-0.133	1.3E-09
Total curvature normalized (t11)	0.006	8.6E-01
Ratio for arc length (t15)	0.033	1.9E-01

The four most correlated traits shown in Fig 1A and 1B and two traits unaffected by age. The full names for tortuosity traits: total squared curvature (t5), total curvature divided over the chord length (t11), and interpolated arc length divided by original arc length (t15). Gender: males and females combined.

Table 3 analyzes the same six features for the AREDS set. The same two Junction traits were observed to change most with age, the number of terminal points (j1, adjusted p-value ~6.5e-15) and the number of bifurcations (j2, adjusted p-value ~3.8e-10). The decline of the values was from 117 to 77 (~34%) for j1 and from 111 to 59 (~47%) for j2, across the AREDS age range of 59 to 84 years (S2 Table).

**Table 3:** Correlation with age for AREDS microvascular traits.

Microvascular trait, Thick	Pearson	p-values adjusted
Number of bifurcation points (j2)	-0.192	3.8E-10
Number of terminal points (j1)	-0.236	6.5E-15
Total squared curvature (t5)	-0.180	3.7E-09
Scale 3 fractal (f3)	0.018	9.9E-01
Total curvature normalized (t11)	-0.009	9.9E-01
Ratio for arc length (t15)	-0.006	9.9E-01

The same traits as in Table 2. Gender: male and females combined.

Thus, both the SardiNIA and AREDS sets showed negative trends for several traits with age. Most clearly, both the observed number of bifurcations of thick vessels (j2) and the number of terminal points in sub-branches (j1) go down with age, and the total squared curvature (t5) shows a trend to relative straightening of the vessels as well. In addition, one fractal trait (f3) showed a significant p-value for decline in SardiNIA, a further indication of lower complexity in the “Thick” microvasculature of older individuals. However, the p-value for trait f3 was 8 orders of magnitude lower than the other 3 traits and was not replicated in AREDS.

### Age signal by CHARM features

We additionally carried out an independent assessment of an age effect on fundus morphology using CHARM features (see Methods) that are general descriptors of a pixel plane (and thus not specific to eye morphology). Of three original color planes in eye images of the SardiNIA set, we selected the green channel and implemented a set of 2,919 CHARM features. The six CHARM features with strongest correlation are given in Table 4, where significance of the age signal in the images is extraordinarily high (on the order of 10^−200^ for six features, all Zernike coefficients (with indices 31, 32, 48, 57, 58, and 62) [31] from images manipulated with wavelet followed by Fourier transforms.

**Table 4:** Correlation with age for SardiNIA CHARM features.

CHARM feature	Pearson	p-values adjusted
Zernike(FFT(WL)) #31	0.607	1.0E-199
Zernike(FFT(WL)) #57	0.607	1.0E-199
Zernike(FFT(WL)) #58	0.604	9.4E-198
Zernike(FFT(WL)) #32	0.598	5.1E-193
Zernike(FFT(WL)) #48	0.595	2.4E-190
Zernike(FFT(WL)) #62	0.593	3.1E-189

The six most correlated features shown (see text). Gender: males and females combined. For these entries, Fast Fourier transform (FFT) and wavelets (WL) [25] were used to transform each image before computing Zernike polynomials.

### Classification analysis

We used classification (Methods) to discriminate between age subcohorts using microvasculature traits for SardiNIA and AREDS data; and in addition, we ran analyses for SardiNIA both with the total sample and with males and females separately. For SardiNIA, we defined a younger subcohort as the range of 20–37 y.o., and an older subcohort as the range of 65–89 y.o.

In contrast to previous analyses, here each participant contributed either one or multiple images. The subcohorts were designed to accentuate age differences using 1,092 of the participants. Among them, of the training set we used 217×2 images (males) and 325×2 images (females), leaving 260 (males) and 290 (females) images for testing. For AREDS, we defined a younger subcohort as the range of 59–69 y.o., and an older subcohort as the range of 76–89 y.o., and here we had for training 731×2 (males) and 943×2 (females) images, while dedicating 580 (males) and 700 (females) images for testing.

Classification accuracy was scored as the frequency of correct calls for age cohorts (Methods). The result of the four classification experiments is given in Table 5. In two-group classification the null value (no accuracy) would be 0.5, whereas the discrimination between the two age cohorts considerably exceeded that noise level. For SardiNIA data, classification accuracy was (left column of Table 5) 0.630 (males) and 0.648 (females); and for AREDS accuracy was (right column of Table 5) 0.578 for males and 0.586 for females). In other words, roughly 63% of men and 65% of women in the SardiNIA cohort are correctly classified in either the younger or older age group using the microvasculature traits. In AREDS, about 58–59% of men and women are correctly assigned to the rightful age group. The slightly higher age signal for females may simply reflect the somewhat larger number of females assessed. Also, the age signal in SardiNIA appears stronger than that in AREDS (see Discussion).

**Table 5:** Accuracy of classification of age in two populations.

	SardiNIA	AREDS
Males	0.630	0.578
Females	0.648	0.586

Two subpopulations used as age cohorts (Methods).

In a further analysis, we used classification to contrast the age signal detected by microvascular and CHARM features in the SardiNIA cohort, with males and females combined. Table 6 compares the age signal in three sets of experiments for three different sets of age cohorts as follows. For the analysis of two subcohorts we used the age ranges defined above; for three subcohorts, we had age ranges of 20–38, 48–56, and 65–89; and for four subcohorts the ranges were 20–32, 36–43, 56–61, and 70–89. For the two and three subcohort analyses, the per-class size of training was the same (453 images), while for four subcohort analysis the per-class size of training was 212 images. The test size was about 780 (two subcohorts), 1,150 (three subcohorts), and 970 (four subcohorts) images. The left entries in Table 6 corresponds to classification of samples with microvascular traits; the right entries, to classification with CHARM features. In all the experiments (see in Table 6) the age signal exceeded the noise floor (0.33 for three age groups and 0.25 for four age groups) indicating the capacity to discriminate. But the classification accuracy using CHARM features was notably higher than that for parameterized microvascular traits (see Discussion).

**Table 6:** Comparing traits to CHARM features in classification of age.

Age cohorts	Microvascular traits	CHARM features
2	0.643	0.930
3	0.419	0.765
4	0.339	0.612

Two, three, and four subpopulations used as age cohorts (Methods). Data: SardiNIA set, males and females combined.

### Tests for correlation of individual traits with standard cardiovascular and metabolic traits

To assess whether the identified age-related changes include a signal correlated with pathology, we performed regression analysis for the microvascular traits, where each trait (y) was regressed with age (A), sex (S) and cardiovascular traits (*x*_1_,*x*_2_,…*x*_8_) as *y* = *A*+*A*^2^+*A*∙*S*+*S*+*x*_1_+*x*_2_+⋯+*x*_8_. Eight cardiovascular/physical traits were considered: systolic and diastolic supine blood pressure (the average of the second and third of three successive measurements), anthropometric data (circumference for waist and hip, weight, and height), body mass index, and coronary carotid artery intima media thickness.

The model in this form tests the association between the microvascular traits and age while adjusting for the change in microvasculature due to age. S3 Table shows beta values, with the sign indicating increase or decrease in the values of the trait, along with p-values for the cardiovascular traits (second column), where only one trait (t1) appears significant. Also tabulated are p-values for age (third column), which are again far stronger for the junction traits and indicate that there is an effect of age independent of the cardiovascular/physical traits (cf. Table 2). We also tested for an effect of sex and found a significant effect for one of 11 analyzed traits (S4 Table). Further analyses might explore sex effects in greater detail; but because sex was included as a covariate, we are showing here the impact of aging independent of any sex effect.

### CHARM correlation with blood pressure

The same regression analysis was performed for CHARM features for the SardiNIA cohort for height and systolic blood pressure, the two traits that had shown the strongest CHARM signal. S5 Table shows adjusted p-values for the trait (second column) and corresponding values for age correlation (third column), adjusted for multiple testing at an FDR cut-off at 0.05 [30]. The correlations with height and blood pressure are more significant than seen with traits (in S3 Table), but these CHARM features were selected on the basis of strong correlations with height and blood pressure, and the correlations of those features with age are far weaker than correlations of other features with age (Table 4), presumably reflecting different intrinsic signals in the retinal photographs.

## Discussion

As the life expectancy of the overall population rises, so does the prevalence of age-related disorders. The eye provides a unique opportunity to non-invasively inspect the status of the smaller vessels and changes to the retinal vasculature have been associated with diseases of high morbidity and mortality, such as hypertension and cardio- and cerebrovascular disorders. In addition, the aging eye is particularly vulnerable, and eye disorders such as diabetic retinopathy and age-related macular degeneration are a major public health concern. Several pathologies and age-related conditions (Alzheimer's disease, dementia, renal diseases, hypertension, etc.) have been reported associated with changes in eye microvasculature. Over the course of the last decade, computer science has generated approaches for non-invasive analysis of vasculature health. These approaches in the future may thus be useful to screen, for example, for risk of cardiovascular or cerebrovascular diseases.

In this study we aimed to characterize the aging retinal vasculature by using computer-assisted feature detection. Both analyses of parameterized measures in segmented microvasculature and CHARM feature analysis of unsegmented retinal images provide information about aging-related changes in the eye. The main inference from the estimates of tortuosity, fractal dimension, and junction lengths and numbers is a progressive loss of complexity with age. It is notable that only a subset of traits showed statistically significant changes. For tortuosity, there is no explicit reason why the t5 trait (total squared curvature, measured with the sampled anchor points) was the most sensitive to age; but Hart et al. [5] reported that t5 is one of best traits for classifying vessel segments, noting that it performed significantly better than total curvature. Fractal measures, which are the least intuitive, also showed the weakest signal, for only one trait (f3) and only in the SardiNIA cohort. Junction analyses showed the sharpest changes, and the simplest to interpret: bifurcations and number of termination points of segments both declined, indicating a net loss of microvasculature as part of the aging process.

Compared to the approach with parameterized microvascular traits, we noted more statistically significant changes with age using the “artificial intelligence” CHARM feature approach, in which 2,919 mathematical algorithmic features were assessed in a training/testing approach to age-delimited subsets of the SardiNIA samples. On the one hand, this result shows that there is more age-dependent signal in the fundus photographs than is seen in the extracted microvasculature; which is also consistent with an analysis of age signal with deep learning trained and tested on a very large sample of over 200,000 retinograms from a number of populations that has been published during the preparation of this report [15]. On the other hand, as is the norm for artificial intelligence-based approaches, the origin of the signal for AI, remains unknown and may include features not just of the microvasculature but of other image components. One hint for possible further analyses comes from the finding that a subset of CHARM features, particularly 6 transforms of Zernike polynomial features, were especially discriminating. The polynomial coefficients are usually considered to provide an incisive signal from overly abundant information [31, 32], and in addition, Zernike polynomials are defined on a circle and might thus be more adapted to round images. It may be possible to gain more information by assessing which portion of the images is giving rise to the strong signal (cf. the study of osteoporosis X-rays in [33]).

The observed correlations are probably underestimates reflecting several limitations in the approach that could be remedied in further studies. First, because we wanted to be able to adapt programs and ensure reproducibility of our results, we used the open source software mlvessel-1.4 of [20], which does not measure the caliber of capillaries directly, although in the future, caliber and vessel abnormalities could be further assessed by the commercially available proprietary SIVA software [3, 34]. Second, mlvessel-1.4 does not extract the smaller capillaries effectively, so that the measures of those capillaries were very noisy. Consequently, the changes seen with Thick elements alone approximated those seen with the entire set of extracted features. Third, the program does not discriminate between arterioles and venules, and one analysis [3] showed that tortuosity was significantly greater (p<0.001) in venules. Thus, more refined extraction of the smaller vessels and discrimination of venules and arterioles can provide a more complete and possibly more accentuated age profile of the changes. Fourth, the necessity to downscale retinogram size (Methods) might decrease the accuracy of segmentation and the resultant values for caliber and local tortuosity. Fifth, although the same microvasculature traits gave an age signals in both AREDS and SardiNIA and both studies showed appreciable accuracy in classification trials, there were quantitative differences: SardiNIA showed lesser rates of decline with age of the significant parameters, and somewhat higher age signals in classification. These differences could result in part from the different sizes of the cohorts studied, but they might also reflect a “batch effect” resulting from differences in photograph protocols or even a difference between the founder Sardinian population compared to the cosmopolitan population sampled by AREDS.

In spite of these limitations, the correlation of, for example, blood pressure with tortuosity in our data is comparable to those observed in earlier studies [35]. Trait j1 for the number of terminal points, which had one of the strongest correlations with age, showed marginally significant associations with blood pressure measures and height, independent of the effect of age.

Selected CHARM features also showed stronger correlation with blood pressure–again in accord with a large study in other populations by deep learning [15]. Possibly a combination of observed features and finer resolution of smaller vessels might correlate even better with features of blood flow and the deep learning signal.

## Conclusions

The simple and accessible method we provide can characterize the retinal microvasculature in populations of various age ranges. The results thus far show that the retinal microvasculature clearly loses complexity with age, an effect that is expected to produce reductions in functionality. In fact, sparser vascular networks were found to correlate with diabetes or higher cardiovascular risk in recent population-based Montrachet study [36], though the effect of age per se in the population was not investigated. Whether trait analyses would be of potential utility for computer-assisted assessments of retinal health in individuals would depend on the extent to which batch effects may affect results, and requires further study. In our study, the comparable changes in microvasculature with age in the SardiNIA and AREDS control cohorts are encouraging. In addition, CHARM features or deep learning could provide an observer-independent examination of fundus photographs for case: control studies of eye diseases, aiding in diagnosis or prognosis (Coletta C. et al are finalizing a study on automating diagnosis of macular degeneration using multimodal machine learning).

## Supporting information

S1 Fig Age distribution in the two cohorts, SardiNIA, 2,280 participants of average age 49.7 y.o. and AREDS, 1,178 participants of average age 68.8 y.o. (TIF)

S2 Fig Randomly selected samples of color eye fundus images (SardiNIA) and corresponding blood vessel maps (segmentation by mlvessel-1.4). (TIFF)

S3 Fig Distribution of microvascular traits (SardiNIA) over participants’ age. Two traits with the best correlation (j1, j2) are marked with red rectangle, another two traits with noticeable correlation (t5, f3) are marked with green rectangles, and the two controls (t11, t15) are marked with blue circles.
(TIFF)

S4 Fig Distribution of microvascular traits (AREDS) over participants’ age. The same traits are marked as in S3 Fig.
(TIFF)

S5 Fig SardiNIA set. **(A)** The same as in S1 Fig. **(B)** Corresponds to ages of 60 and above (solid: 60:66.5y.o., dashed: >66.5 y.o.).
(TIFF)

S1 Table SardiNIA set: Change of traits with age. Gender: males and females combined. Age range for SardiNIA set is from 20.3 to 88.4 years. The same six microvascular traits as in Table 1A.
(DOCX)

S2 Table AREDS set: Change of traits with age. The same traits as in S1 Table, but for age range 59 to 84. (The values in italic: the change goes beyond the second decimal.)
(DOCX)

S3 Table Correlation with BP and age for microvascular traits. Correlation was via regression analysis (see text), reported values are beta and FDR for CV traits and age. Data: SardiNIA set, males and females combined.
(DOCX)

S4 Table Correlation with sex and age for microvascular traits. The same analysis as in S3 Table, reported values are beta and FDR for sex and age.
(DOCX)

S5 Table Correlation with BP and age for CHARM features. The same analysis as in S3 Table but for the CHARM features here. Data: SardiNIA set, males and females combined. Note that the CHARM features selected here for top p-values for height and blood pressure, are not the same as the ones in Table 1C, selected for maximum p-value for age differences.
(DOCX)

S1 Appendix Equations and descriptions for two common vessel traits, tortuosity (Part A) and fractals (Part B).
(PDF)

## Abbreviations

AREDS: Age-Related Eye Disease Study
CHARM: Compound Hierarchy Of Algorithms Representing Morphology
SVM: Support Vector Machines
WND: Weighted Neighbor Distances
