# Supplementary figures and images for: Age-related changes of the retinal microvasculature
Source: PLoS One. 2019 May 2;14(5):e0215916. doi: 10.1371/journal.pone.0215916 (PMC6497255; doi:10.1371/journal.pone.0215916)

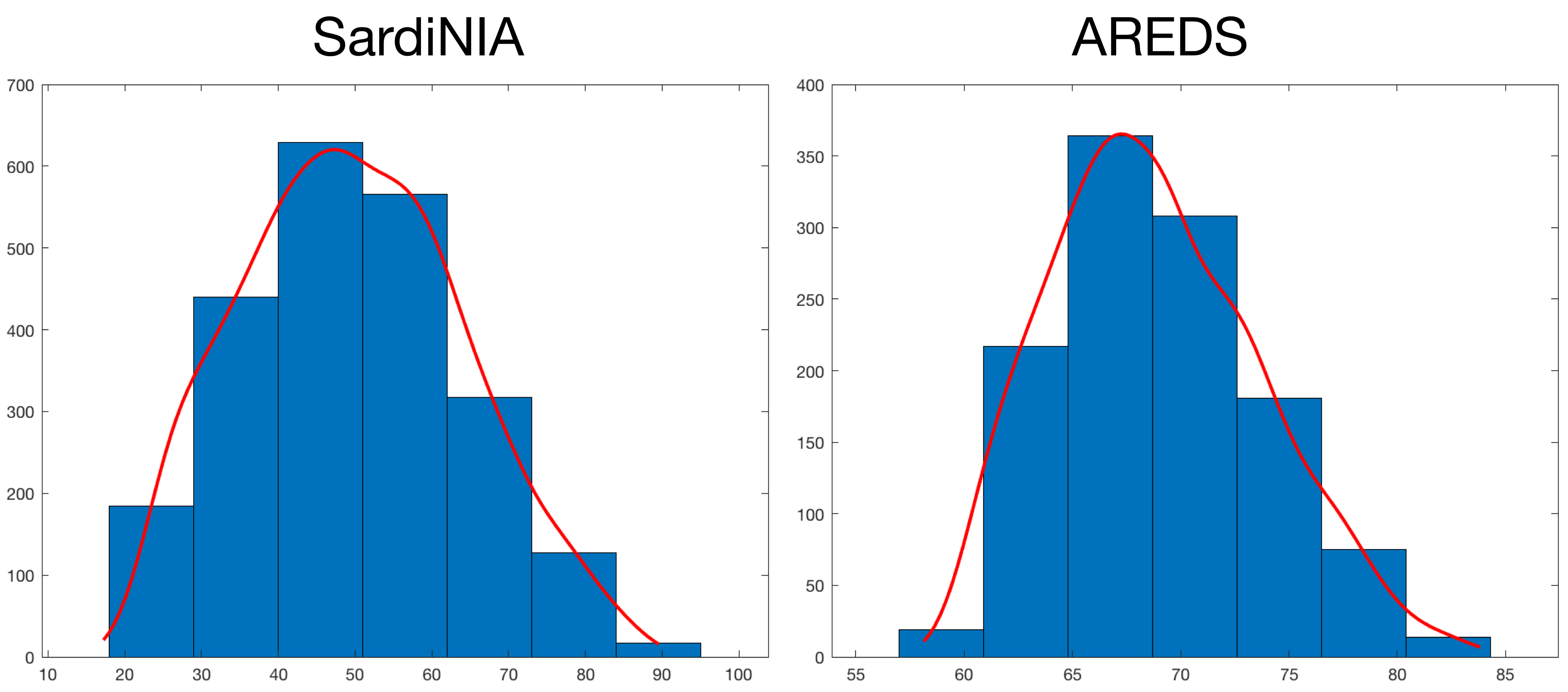

Supplement: S1 Fig — (TIF) [file pone.0215916.s001.tif]

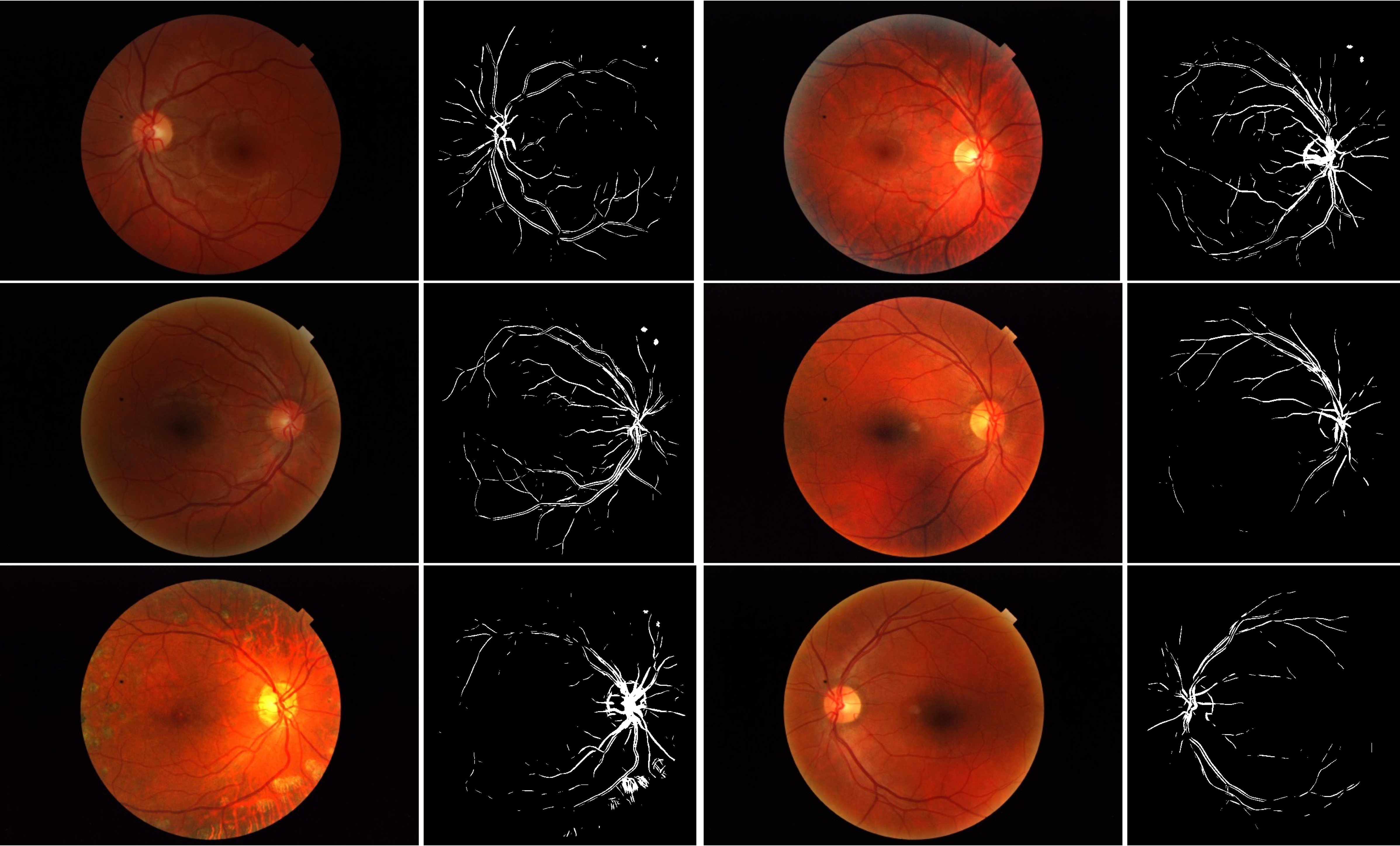

Supplement: S2 Fig — (TIFF) [file pone.0215916.s002.tiff]

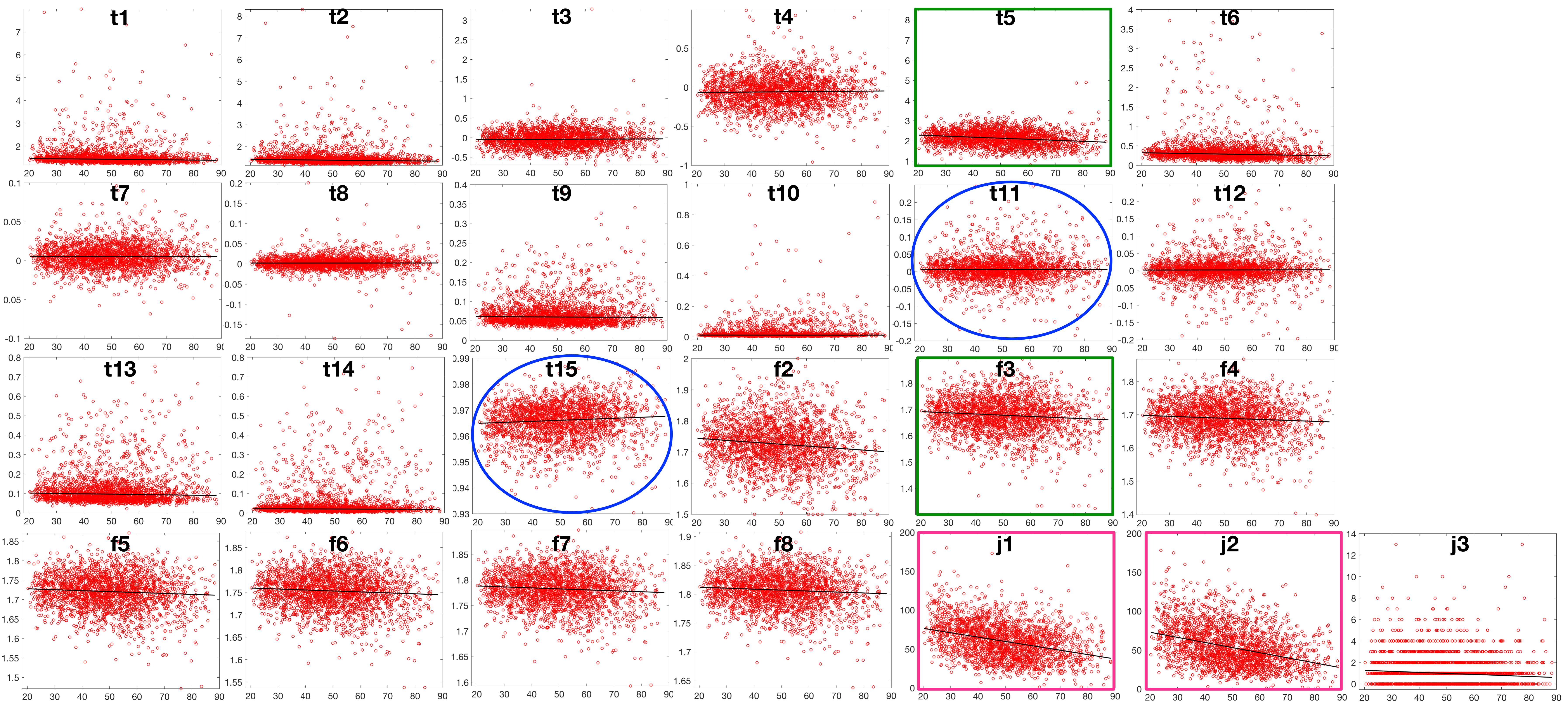

Supplement: S3 Fig — Two traits with the best correlation (j1, j2) are marked with red rectangle, another two traits with noticeable correlation (t5, f3) are marked with green rectangles, and the two controls (t11, t15) are marked with blue circles. (TIFF) [file pone.0215916.s003.tiff]

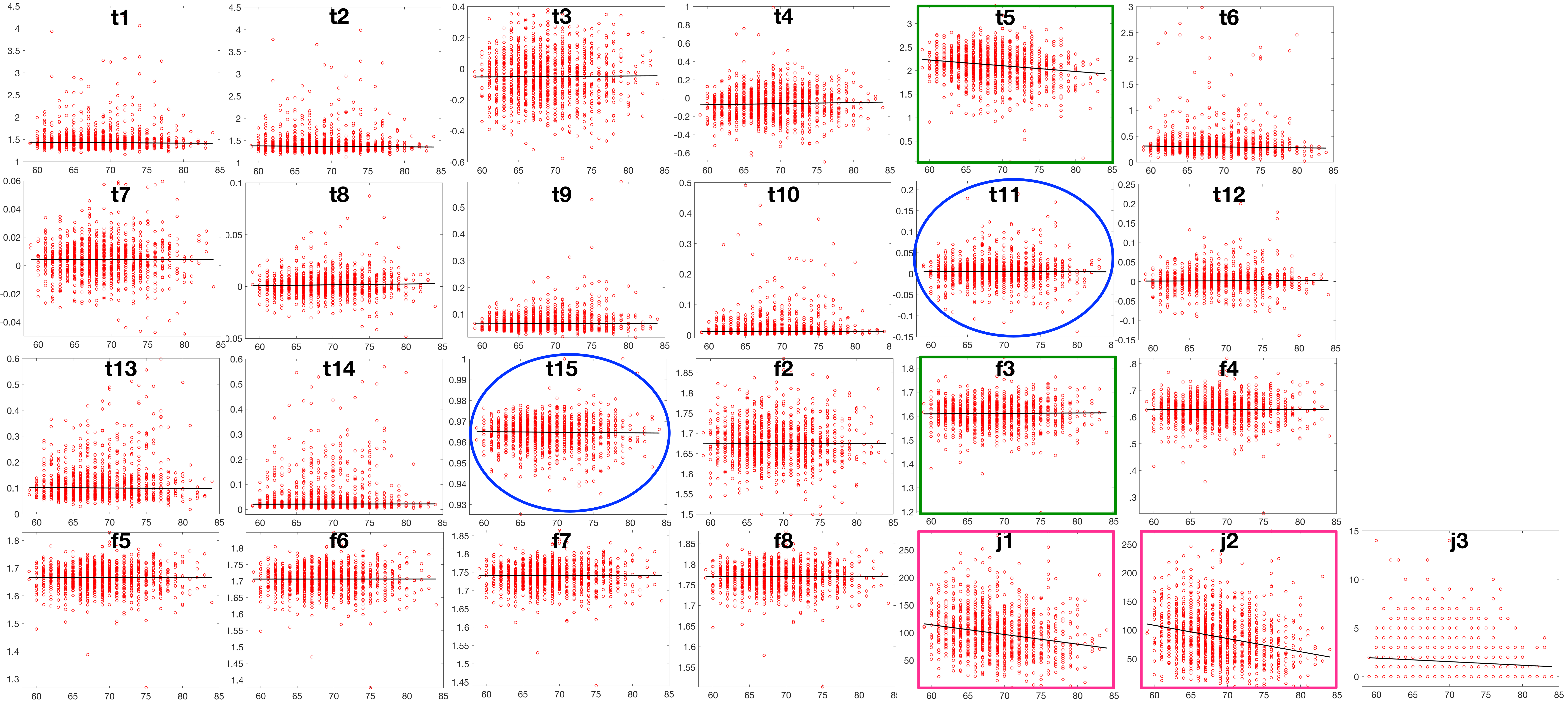

Supplement: S4 Fig — The same traits are marked as in S3 Fig. (TIFF) [file pone.0215916.s004.tiff]

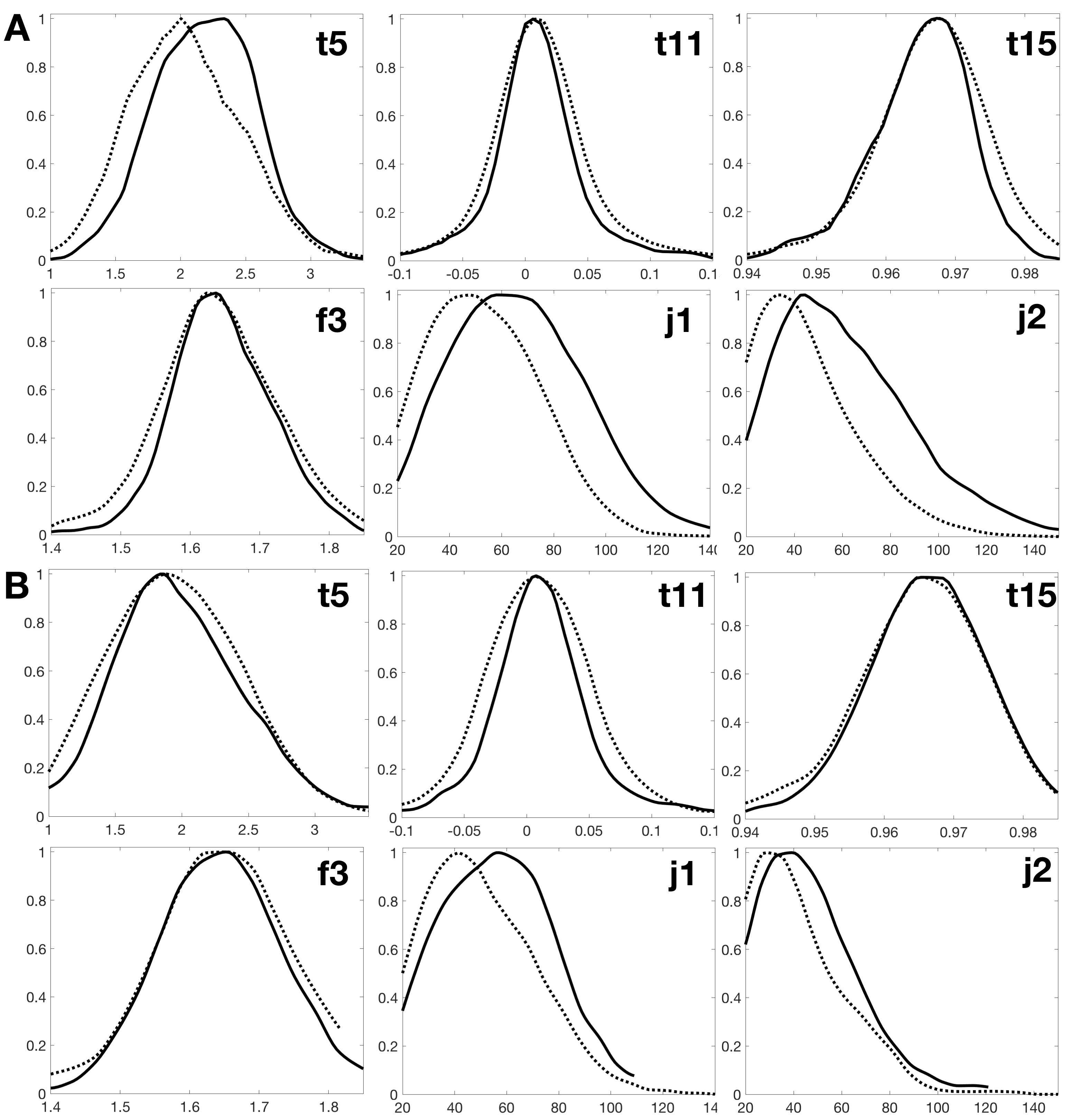

Supplement: S5 Fig — (A) The same as in S1 Fig. (B) Corresponds to ages of 60 and above (solid: 60:66.5y.o., dashed: >66.5 y.o.). (TIFF) [file pone.0215916.s005.tiff]
