# Supplementary material for: Age-related changes of the retinal microvasculature
Source: PLoS One. 2019 May 2;14(5):e0215916. doi: 10.1371/journal.pone.0215916 (PMC6497255; doi:10.1371/journal.pone.0215916)
Supplement: S1 Table — Gender: males and females combined. Age range for SardiNIA set is from 20.3 to 88.4 years. The same six microvascular traits as in Table 1A. (DOCX) [file pone.0215916.s006.docx]

**S1 Table.** SardiNIA set: change of traits with age.

| Microvascular trait, Thick | Value at  20.3 y.o. | Value at  88.4 y.o. | 10 year  change | 10 year  change, % |
| --- | --- | --- | --- | --- |
| Number of bifurcation points (j2) | 74.07 | 31.29 | -6.28 | -8.5 |
| Number of terminal points (j1) | 76.88 | 40.59 | -5.33 | -6.9 |
| Total squared curvature (t5) | 2.27 | 1.98 | -4.21E-2 | -1.9 |
| Scale 3 fractal (f3) | 1.69 | 1.65 | -6.39E-3 | -0.4 |
| Total curvature normalized (t11) | 9.06E-3 | 1.04E-2 | 2.02E-4 | ~0 |
| Ratio for arc length (t15) | 9.65E-1 | 9.66E-1 | 1.84E-4 | ~0 |

Gender: males and females combined. Age range for SardiNIA set is from 20.3 to 88.4 years. The same six microvascular traits as in Table 1A.
