# Supplementary material for: Age-related changes of the retinal microvasculature
Source: PLoS One. 2019 May 2;14(5):e0215916. doi: 10.1371/journal.pone.0215916 (PMC6497255; doi:10.1371/journal.pone.0215916)
Supplement: S2 Table — The same traits as in S1 Table, but for age range 59 to 84. (The values in italic: the change goes beyond the second decimal.) (DOCX) [file pone.0215916.s007.docx]

**S2 Table.** AREDS set: change of traits with age.

| Microvascular trait, Thick | Value at  59 y.o. | Value at  84 y.o. | 10 year  change | 10 year  change, % |
| --- | --- | --- | --- | --- |
| Number of bifurcation points (j2) | 110.79 | 58.60 | -20.87 | -18.8 |
| Number of terminal points (j1) | 117.23 | 77.16 | -16.03 | -13.7 |
| Total squared curvature (t5) | 2.23 | 1.91 | -1.28E-1 | -5.8 |
| Scale 3 fractal (f3) | *1.61* | *1.61* | 1.96E-3 | ~0 |
| Total curvature normalized (t11) | 6.84E-3 | 5.34E-3 | -5.99E-4 | -8.8 |
| Ratio for arc length (t15) | 9.65E-1 | 9.64E-1 | -7.60E-5 | ~0 |

The same traits as in S1 Table, but for age range 59 to 84. (The values in italic: the change goes beyond the second decimal.)
