# Supplementary material for: Age-related changes of the retinal microvasculature
Source: PLoS One. 2019 May 2;14(5):e0215916. doi: 10.1371/journal.pone.0215916 (PMC6497255; doi:10.1371/journal.pone.0215916)
Supplement: S3 Table — Correlation was via regression analysis (see text), reported values are beta and FDR for CV traits and age. Data: SardiNIA set, males and females combined. (DOCX) [file pone.0215916.s008.docx]

**S3 Table.** Correlation with BP and age for microvascular traits.

| Microvascular trait | C.V. trait (beta, FDR) | Age (beta, FDR) |
| --- | --- | --- |
| Ratio of arc and chord lengths (t1) | Systolic (-3.91E-03, 3.73E-02) | -2.25E-03, 9.26E-01 |
| Ratio for arc lengths (t15) | Hip ( 1.42E-04, 1.86E-01) | 8.26E-05, 9.26E-01 |
| Ratio for arc lengths (t15) | Systolic (-3.31E-05, 3.82E-01) | 8.26E-05, 9.26E-01 |
| Number of terminal points (j1) | Systolic (-9.58E-02, 3.82E-01) | -1.93E+00, 3.65E-11 |
| Number of bifurcation points (j2) | Systolic (-9.53E-02, 3.82E-01) | -1.91E+00, 8.54E-10 |

Correlation was via regression analysis (see text). Data: SardiNIA set, males and females combined.
