# Supplementary material for: Age-related changes of the retinal microvasculature
Source: PLoS One. 2019 May 2;14(5):e0215916. doi: 10.1371/journal.pone.0215916 (PMC6497255; doi:10.1371/journal.pone.0215916)
Supplement: S4 Table — The same analysis as in S3 Table, reported values are beta and FDR for sex and age. (DOCX) [file pone.0215916.s009.docx]

**S4 Table.** Correlation with sex and age for microvascular traits.

| Microvascular trait | Sex (beta, FDR) | Age (beta, FDR) |
| --- | --- | --- |
| Ratio of arc and chord lengths (t1) | -6.67E-02, 7.08E-01 | -2.25E-03, 9.10E-01 |
| Total curvature (t3) | 2.25E-02, 7.08E-01 | 7.80E-04, 9.10E-01 |
| Total squared curvature (t5) | -2.04E-02, 8.82E-01 | 4.23E-03, 8.71E-01 |
| Total curvature, arc normalized (t7) | -6.92E-06, 9.98E-01 | 1.59E-04, 8.71E-01 |
| Total curvature, chord normalized (t11) | -7.95E-03, 6.20E-01 | 2.44E-04, 9.10E-01 |
| Ratio for arc lengths (t15) | -1.58E-03, 6.20E-01 | 8.26E-05, 8.71E-01 |
| Box counts, scale 2 (f2) | -1.33E-02, 6.20E-01 | -1.36E-05, 9.91E-01 |
| Box counts, scale 3 (f3) | -1.86E-02, 5.61E-01 | -9.77E-06, 9.91E-01 |
| Box counts, scale 4 (f4) | -1.48E-02, 5.61E-01 | -2.13E-04, 9.10E-01 |
| Number of terminal points (j1) | 1.78E+01, 1.69E-03 | -1.93E+00, 3.98E-11 |
| Number of bifurcation points (j2) | 1.40E+01, 3.33E-02 | -1.91E+00, 9.32E-10 |

Correlation was via regression analysis (see text). Data: SardiNIA set, males and females combined.
