# Supplementary material for: Age-related changes of the retinal microvasculature
Source: PLoS One. 2019 May 2;14(5):e0215916. doi: 10.1371/journal.pone.0215916 (PMC6497255; doi:10.1371/journal.pone.0215916)
Supplement: S5 Table — The same analysis as in S3 Table but for the CHARM features here. Data: SardiNIA set, males and females combined. Note that the CHARM features selected here for top p-values for height and blood pressure, are not the same as the ones in Table 1C, selected for maximum p-value for age differences. (DOCX) [file pone.0215916.s010.docx]

**S5 Table.** Correlation with BP and age for CHARM features.

| CHARM feature | C.V. trait (adjusted p-value) | Age (beta, FDR) |
| --- | --- | --- |
| EdgeFeatures #23 | Height (-5.80E+00, 1.53E-06) | -1.61E+0, 5.18E-02 |
| CombMoments(Cheb(FFT)) #30 | Systolic (-3.22E-02, 5.59E-03) | 6.60E-2, 1.14E-01 |
| Zernike() #45 | Systolic ( 1.66E-04, 5.59E-03) | 2.04E-4, 3.80E-01 |
| Zernike() #15 | Systolic ( 7.47E-06, 1.08E-02) | -2.12E-6, 8.62E-01 |
| TamuraTextures() #3 | Systolic ( 2.86E-05, 1.16E-02) | -9.84E-5, 1.07E-02 |

The same analysis as in S3 Table but for the CHARM features here. Data: SardiNIA set, males and females combined. Note that the CHARM features selected here for top p-values for height and blood pressure, are not the same as the ones in Table 1C, selected for maximum p-value for age differences.
