# Supplementary material for: Age-related changes of the retinal microvasculature
Source: PLoS One. 2019 May 2;14(5):e0215916. doi: 10.1371/journal.pone.0215916 (PMC6497255; doi:10.1371/journal.pone.0215916)
Supplement: S1 Appendix — Equations and descriptions for two common vessel traits, tortuosity (Part A) and fractals (Part B). (PDF) [file pone.0215916.s011.pdf]

Appendix A: Tortuosity traits

Let $\vec{\gamma}$ be a $\mathbb{R}^2$ curve: $\overrightarrow{\gamma(t)} = \begin{bmatrix} x(t) \\ y(t) \end{bmatrix}$, where t is a parameter defined on a segment $t \in [a, b]$.

Then the curve's arc and chord lengths are

$\mathcal{L}_{\mathcal{A}}(\gamma) = \int_a^b \left\| \overrightarrow{\gamma'(t)} \right\| dt$, and $\mathcal{L}_{\mathcal{C}}(\gamma) = \left\| \overrightarrow{\gamma(a)} - \overrightarrow{\gamma(b)} \right\|$, respectively, and $\overrightarrow{\gamma'(t)} = \begin{bmatrix} x'(t) \\ y'(t) \end{bmatrix}$.

The ratio of the curve's arc and chord lengths:

$T_1 = \mathcal{L}_{\mathcal{A}}(\gamma)/\mathcal{L}_{\mathcal{C}}(\gamma)$

The curvature of the curve $\vec{\gamma}$ is $\kappa(t) = \frac{x'(t)y''(t) - x''(t)y'(t)}{\left\| \overrightarrow{\gamma'(t)} \right\|^3}$.

The total curvature over the whole segment and total squared curvature, respectively:

$T_2 = \int_a^b \kappa(t) dt, \quad T_3 = \int_a^b \kappa^2(t) dt.$

The total curvature and total squared curvature are normalized by the curve's arc length:

$T_4 = \int_a^b \kappa(t) dt / \mathcal{L}_{\mathcal{A}}(\gamma), \quad T_5 = \int_a^b \kappa^2(t) dt / \mathcal{L}_{\mathcal{A}}(\gamma).$

The same measures are normalized by the curve's chord lengths:

$T_6 = \int_a^b \kappa(t) dt / \mathcal{L}_{\mathcal{C}}(\gamma), \quad T_7 = \int_a^b \kappa^2(t) dt / \mathcal{L}_{\mathcal{C}}(\gamma).$

NB: The given set of measures was computed for the given curves at the set of points defined by given resolution of the vessel branch. The skeleton for the vessel map as computed by the segmentation algorithm might turn out noisy. A smoothing approximation of the branch contour with an interpolated set of contour coordinates was implemented. Thus, each tortuosity trait (T_1 through T_7) was computed for sampled and smoothed coordinates.

Another feature weights smoothed (interpolated) value against sampled value for the curve's arc:

$T_{\text{ratio}} = \mathcal{L}_{\mathcal{A}}(\gamma_{\text{interp}})/\mathcal{L}_{\mathcal{A}}(\gamma_{\text{samp}})$

Appendix B: Fractal traits

Per-scale vectors: box size ($s = [1024\ 512\ \dots\ 1]$), resolution ($r = 1/s$), normalized resolution ($x = r/r_1$), box counts (c), and FD trait vector ($y = \log c / \log x$). Vectors have dimension of 8, but normalized resolution in the first scale is 1, and the FD trait in the first scale is not defined and never used.
